# Case report: Cholesterol granuloma of femur

**DOI:** 10.3389/fsurg.2022.944499

**Published:** 2022-12-07

**Authors:** Shenshen Hao, Shengli Dong, Hongke Li, Shuai Liu, Honglei Chen, Zhifang Zhang

**Affiliations:** Department of Spine and Bone Oncology, General Hospital of Pingmei Shenma Medical Group, Pingdingshan, China

**Keywords:** cholesterol granuloma, femur, benign lesion, surgical treatment, case report

## Abstract

**Background:**

Cholesterol granuloma (CG) is a particular type of granulation tissue reaction. It is a very rare benign lesion characterized by swelling growth due to a large number of cholesterol crystals and foreign body giant cells. There has been no report of a CG of the femur.

**Case presentation:**

A 74-year-old woman suffered from pain and discomfort in the upper right knee for 10 years, which became aggravated for 10 days. She was diagnosed with CG of the right femur at our hospital and was treated with surgery. During the operation, a large amount of yellow-brown, oily, crystal structures was found. Postoperative pathology investigations confirmed the lesion as a CG. Postoperative follow-up was carried out for 15 months that confirmed the effect of treatment was satisfactory.

**Conclusions:**

CG of the femur is an extremely rare benign lesion. Surgical treatment can provide effective treatment results.

## Introduction

Cholesterol granuloma (CG) was a benign lesion with swelling growth (1). Multiple parts of the body might appear, and the systemic incidence rate was 1/300,000–1/200,000, of which temporal bone was the most common (2). According to some sporadic reports, CG could be formed in various parts, such as thyroid (3), lung (4), breast (5), mediastinum (6), testis (7), pancreas (8), eyes (9), kidney (10), peritoneum (11), lymph nodes (12), petrous apex (13) and facial bones (14).

CG was rarely reported in the bones of the limbs, and some scholars have reported case reports in the humerus (15) and radius (16). There has been no report of CG in the femur. We admitted a patient with CG of the femur and treated it with surgery, and followed up for 15 months after the operation. Given its rarity, we reported on it for the first time to get a preliminary understanding of its pathological characteristics and its occurrence process. We hope that it may provide some reference to scholars who study CG.

## Case presentation

**Figure 1:**
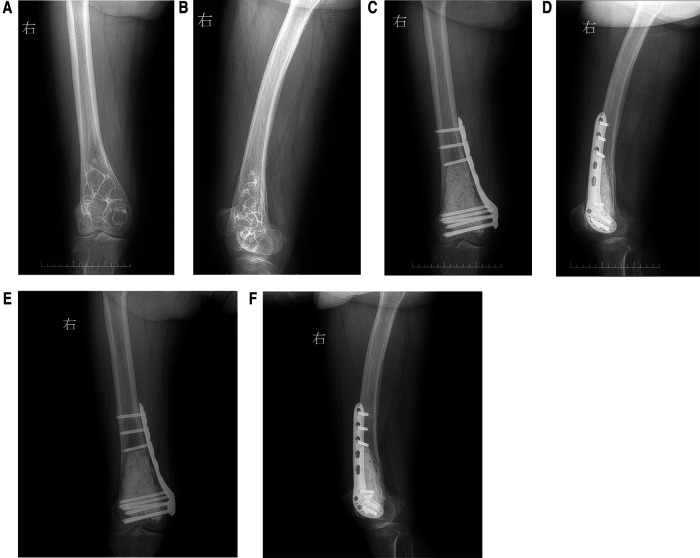
(**A**) preoperative anterior x-ray of the right femur was a multilocular cystic low-density focus with hardened edges; (**B**) preoperative lateral x-ray of the right femur; (**C**) first week of the postoperative anterior x-ray of the right femur the bone morphology of the distal right femur was satisfactory, and the internal fixation position was satisfactory; (**D**) first week of the postoperative lateral x-ray of the right femur the bone morphology of the distal right femur was satisfactory, and the internal fixation position was satisfactory; (**E**) 15 months of the postoperative anterior x-ray of the right femur showed that the fusion of the distal right femur was satisfactory, the bone quality was unchanged, the shape was satisfactory, and the internal fixation position was good; (**F**) 15 months of the postoperative lateral x-ray of the right femur showed that the fusion of the distal right femur was satisfactory, the bone quality was unchanged, the shape was satisfactory, and the internal fixation position was good.

**Figure 2:**
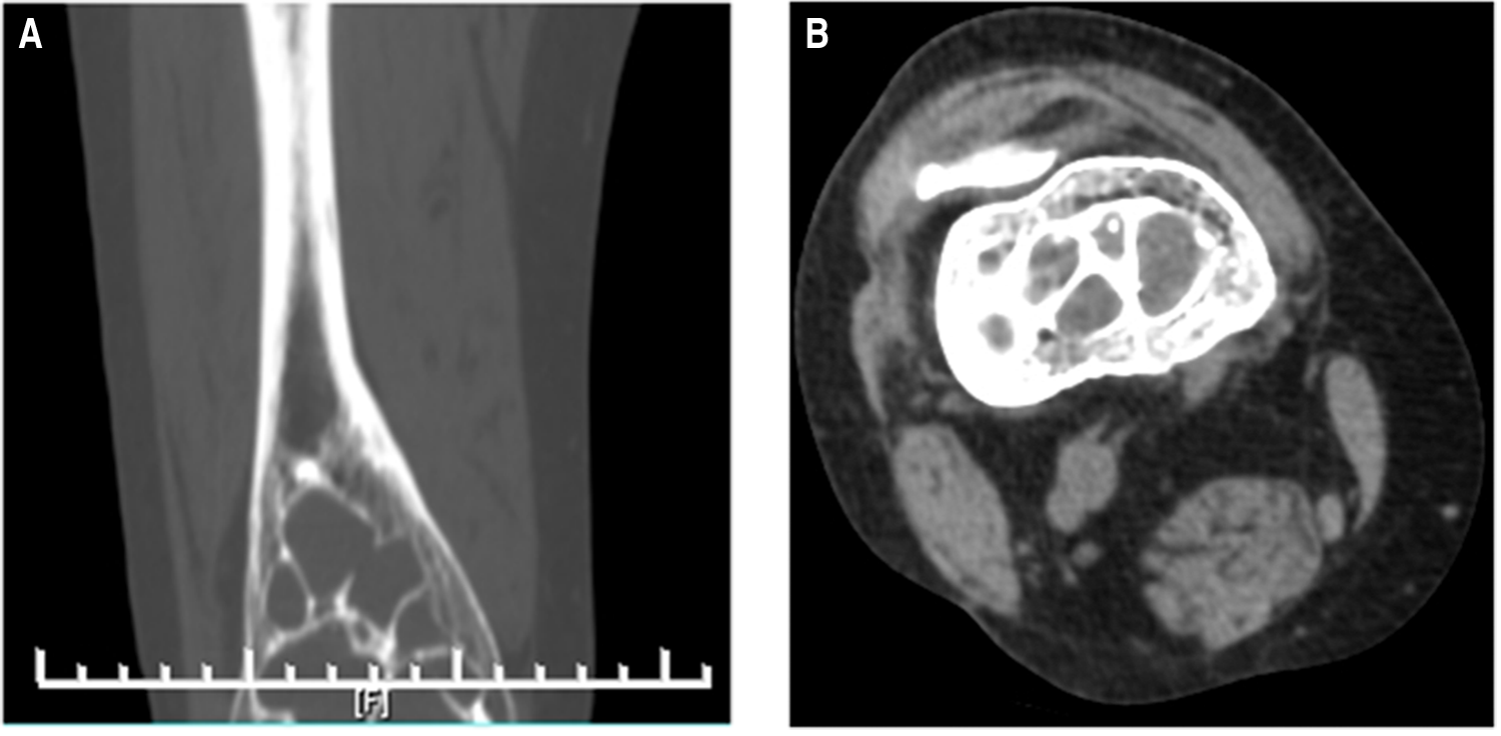
(**A**) preoperative forntal CT indicating that there were multiple cystic low-density shadows in the lower part of the right femur, with clear borders; (**B**) preoperative horizontal cross-sectional CT indicating that there were multiple cystic low-density shadows in the lower part of the right femur, with clear borders, sclerosis margins around, and no obvious abnormalities in adjacent soft tissues.

**Figure 3:**
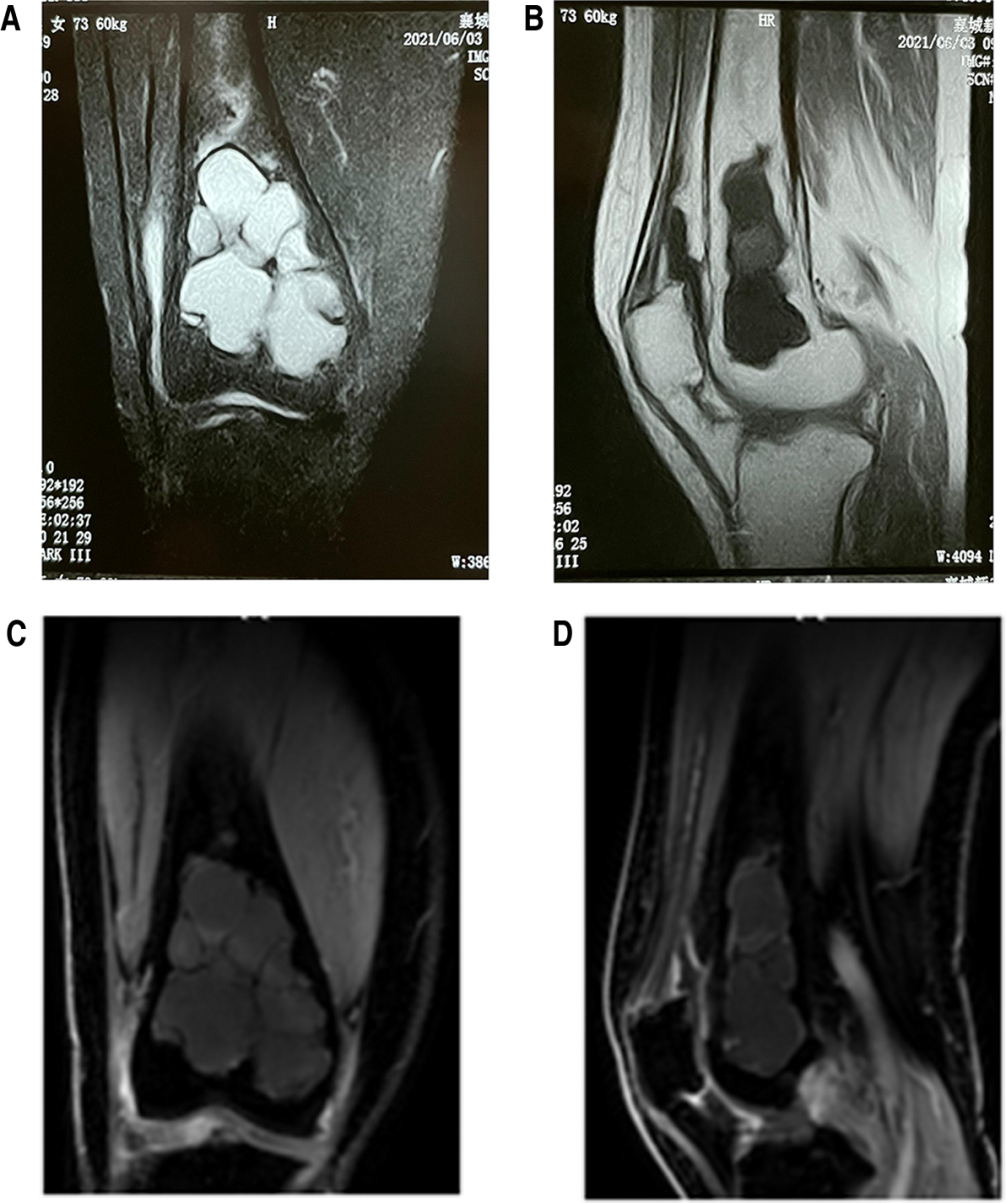
(**A**) preoperative frontal MRI indicating multiple cystic abnormal signal shadows in the lower right femur and the surrounding soft tissues had no special changes; (**B**) preoperative sagittal cross-sectional MRI indicating multiple cystic abnormal signal shadows and the surrounding soft tissues had no special changes; (**C**) preoperative frontal contrast-enhanced MRI indicating multiple cystic abnormal signal shadows and the surrounding soft tissues had no special changes; (**D**) preoperative sagittal cross-sectional contrast-enhanced MRI indicating multiple cystic abnormal signal shadows and the surrounding soft tissues had no special changes.

A 74-year-old female patient, height 160 cm, weight 60 kg, was admitted to our hospital with the chief complaint of “right knee pain and discomfort for 10 years, and aggravation for 10 days.” The patient had no apparent cause to experience right knee pain 10 years ago, slightly restricted activity, no weakness of lower limbs. First, after a rest, her symptoms persisted. Then, she received symptomatic and conservative treatments such as anti-inflammatory and pain relief, physical therapy, etc. The specifics were unknown, and the symptoms were reduced. Symptoms recurred during the period. Until 10 days ago, her pain symptoms were repeated and significantly worse than before, accompanied by limited mobility. Conservative treatment was still chosen, and the symptoms did not alleviate. The physical examination revealed that the skin of the right knee was intact and not damaged, and there was no redness, swelling or edema. There was tenderness and percussion pain in the right knee, without obvious abnormality. The pain of visual analogue scale (VAS) was 6 scales. The hospital for special surgery (HSS) scales was 57 scales. X-ray revealed that the distal end of the right femur was a multilocular cystic low-density focus with hardened edges (Figures 1A,B). CT revealed multiple cystic low-density shadows in the lower part of the right femur, with clear borders, sclerosis margins around, and no apparent abnormalities in adjacent soft tissues (Figures 2A,B). MRI and contrast-enhanced MRI examination revealed multiple cystic abnormal signal shadows in the lower right femur, and the surrounding soft tissues had no notable changes (Figures 3A–D). Laboratory examination results showed that red blood cells were 3.85 × 10^12^/L, cholesterol was 5.22 mmol/L, prothrombin time was 12.20s, prothrombin time activity ratio was 88.00%, international normalized ratio was 1.14, activated partial thromboplastin time was 24.70s, and thrombin time was 16.20s. These indicators were all within the normal range. She had a history of hypertension for 20 years, took 20 mg of “nifedipine sustained-release tablets” orally once a day. The blood pressure was controlled within the normal range. She also had a history of hyperthyroidism for more than 10 years. 3 months ago, she took 10 mg of “methimazole tablets” orally three times a day and changed to twice a day with the same dose one and a half months ago. The thyroid function was in the normal range. Therefore, our diagnosis was 1. A lesion suggestive of a CG of the right femur, 2. Right knee osteoarthritis, 3. Hypertension, 4. Hyperthyroidism.

**Figure 4:**
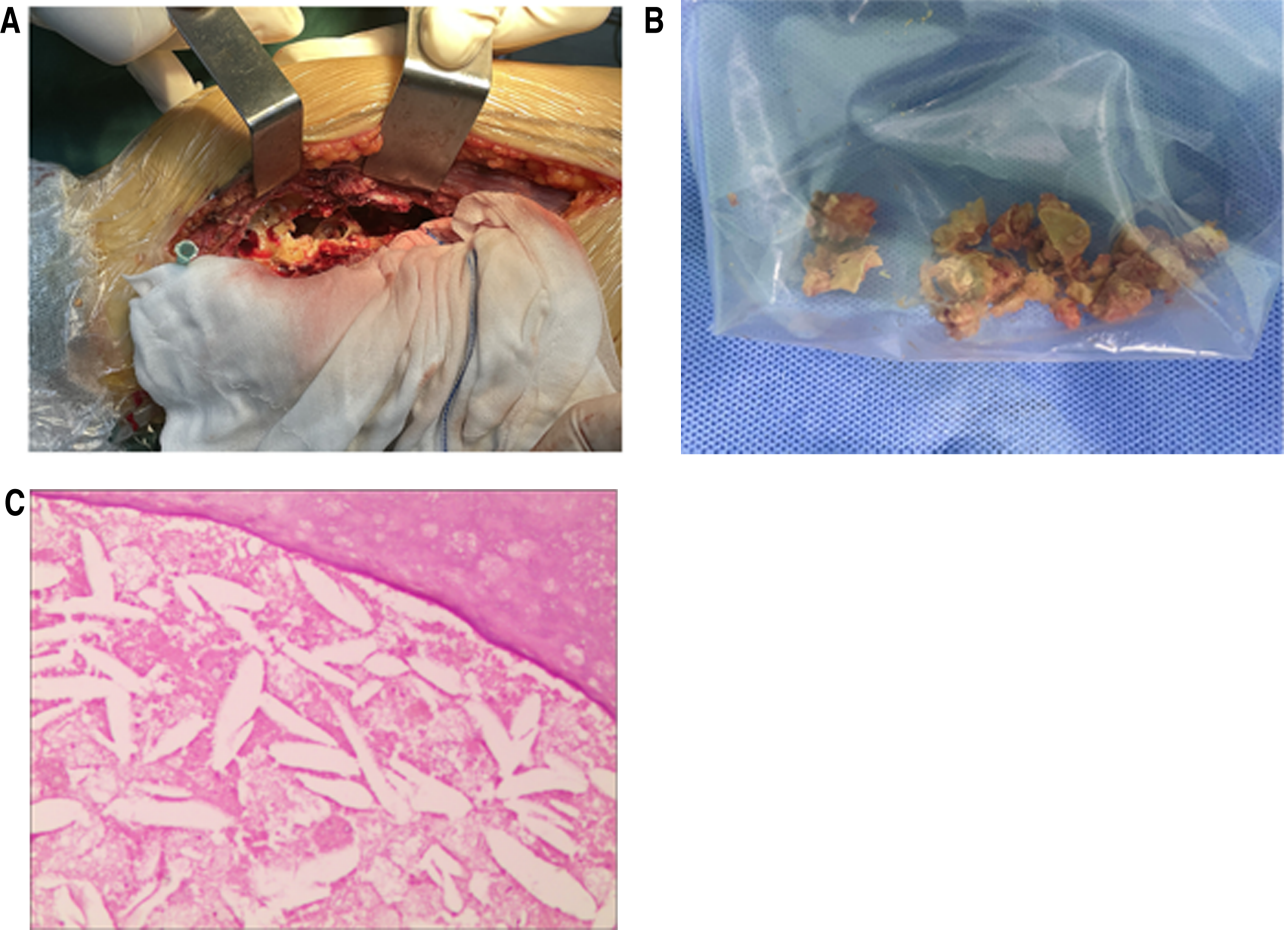
(**A**) after the femur was opened, the bone marrow cavity was filled with CG; (**B**) a part of CG removed from the right femur; (**C**) postoperative pathological examination film, under the microscope with 100 times, a large number of cholesterol crystals were seen in it.

The patient underwent surgery after complete preoperative preparation. It was divided into two main steps. The first was to take a left iliac bone for intraoperative bone grafting. The second was surgery on the part of the femur where CG lesions occurred. The specific steps of the latter were as follows. A longitudinal 15 cm incision was made on the inner side of the distal right thigh. The position of the knee joint space was given priority to determine via a syringe needle to protect the joint capsule. Then the medial condyle of the distal femur and the distal third of the femoral shaft was exposed. Gauze soaked in distilled water was placed around the incision to prevent the diseased tissue from contaminating the normal tissue. A window of approximately (2∼4)cm × 7 cm was opened on the medial cortical bone corresponding to the CG lesion. A large number of yellow-brown oily crystal structures could be seen(Figures 4A,B). The diseased bone was scraped off, then the edge was burnt by electric knife with spray coagulation mode (80 Joule units). 80 degrees distilled water was used to rinse the marrow cavity and bone wall. The above process was alternated three times to achieve the effect of killing diseased cells. The ilium taken in the first step was implanted near the articular surface of the CG lesion to increase the bone mass in the femoral condyle area, and the remaining cavity was filled with high-viscosity bone cement. An anatomical titanium plate was placed and fixed. The wound placed a drainage tube, was sutured and bandaged under pressure.

The pathological examination of the diseased tissue showed that new bone was seen under the microscope, cystic degeneration was seen around it, powder-stained no structure was seen in the cyst cavity, and a large number of cholesterol crystals were seen in it (Figure 4C). X-ray of the right femur was performed on the first week after the operation, the pain of VAS declined to 3 scales, and the results showed that the bone morphology of the distal right femur was satisfactory, and the internal fixation position was satisfactory (Figure 1C,D). X-ray was performed at the third month of postoperative follow-up, and the results showed that the bone quality of the distal right femur was unchanged, the shape was satisfactory, and the internal fixation position was fine. At this time, the patient's right knee pain symptoms and restricted activity had been significantly improved. After 15 months of follow-up, x-ray films were reviewed, and the results showed that the fusion of the distal right femur was satisfactory, the bone quality was unchanged, the shape was satisfactory, and the internal fixation position was good (Figure 1E,F). The right knee joint had no pain and could move freely. The VAS pain was reduced to grade 1 scale, the HSS scales was 85 scales. and the operation effect was good.

## Discussion

CG is a term of histology, which is a particular type of granulation tissue reaction. It is results from the body's cellular response to cholesterol crystal foreign bodies produced by the decomposition of blood and local tissues. CG is often yellowish-brown sludge under naked eye observation. A large number of cholesterol crystals with diamond-shaped fissures could be seen under microscopy. There are foreign body multinucleated giant cells, macrophages, tissue cells, and a large number of lymphocytes, fibrin, and large blood vessels, and there are black particles of hemosiderin in between. Because this blood vessel is easy to rupture, fresh or old bleeding can be seen at the same time. Therefore, CG lesions were characterized by rhomboid or rhombic cholesterol crystals in chronic inflammatory granulation tissue, surrounded by foreign body giant cells, often fresh or old bleeding, accompanied by hemosiderin (17).

Since CG is a sporadic disease, the current research reports mostly case reports. Among them, the most common report of CG was related to petrous apex, so the hypothesis of its pathogenesis was also based on it. There were two main types: the classic obstruction-vacuum hypothesis and the exposed marrow hypothesis (18). Zheng Wenkui et al. reported a case of CG of the humerus and tried to give a preliminary description of its possible formation process (15). Since both the humerus and femur belong to the upper bone of the limbs, they may have certain similarities in structure and function. However, the specific mechanism of formation of CG of the femur is still unknown.

In general, there are two important factors in the formation of CG. On the one hand, the formation of CG could not be separated from the accumulation of cholesterol. The yellow bone marrow of adults is rich in fat tissue that breaks down to produce cholesterol. This undoubtedly provides a prerequisite for the formation of CG. On the other hand, the formation of CG is also closely related to blood which provides a good source of cholesterol. Generally, when cholesterol crystals appeared as a foreign body, the body's immune response was that phagocytes engulfed it. Therefore, under normal circumstances, a small number of cholesterol crystals were produced, and CG was not formed. However, when the rate of production of cholesterol crystals increased, and the output rate exceeded the speed of the body's phagocytosis, this situation would inevitably lead to the accumulation of cholesterol crystals, resulting in a continuous increase in its volume. At this time, the body's response was to unite the phagocytic cells to form a new type of cell, that was, the foreign body giant cell. When an increasing number of these new cells slowly surrounded the cholesterol crystals, their phagocytosis became relatively weak, to some extent.

The formation of CG was often accompanied by blood vessel proliferation, and most of these blood vessels were pathological types of blood vessels, which were easy to rupture and bleeding; bleeding led to the continuous increase of cholesterol content, while red blood cells continued to degenerate and decompose to produce cholesterol crystals, which stimulated the foreign body, leading to further aggravation of bleeding and inflammation; eventually, a hemorrhage-inflammation malignant circulatory system was formed (19). Theoretically, patients with CG should have higher serum cholesterol levels than average. However, by consulting the reported cases in the literature, the patient's serum cholesterol was in the normal range, and there was no systemic bleeding factor (20). Our patient's serum cholesterol was also in the normal range. Therefore, we hypothesized that there was a possibility that the vital role in the formation of CG was the ability to produce cholesterol locally in the lesion, and it was not directly related to serum cholesterol content or coagulation disorders.

CG was also a disease that was not easily detected because of its milder clinical symptoms and slow progress. Therefore, when CG occurred in the distal femur, the initial signs were mainly mild pain, which had similar symptoms to common knee arthritis, and there is no abnormality in the early x-ray, which is easy to be misdiagnosed or missed. This also makes it difficult to differentiate CG from some common benign bone diseases. However, MRI had certain advantages in distinguishing normal tissues from diseased tissues. CG had a special performance on MRI images with diagnostic and differential diagnosis value, such as T1 and T2 weighted images-high signal and no enhancement or only slight peripheral enhancement signal (21). Therefore, it was a wise choice to perfect the relevant examination of MRI when the disease could not be clearly defined. Generally speaking, the pathological examination was the most critical examination for diagnosing the CG, and a large number of cholesterol crystals could be seen. A needle biopsy before surgery is necessary to help diagnose the disease. However, no preoperative needle biopsy was performed in this case. The reason is that we have discussed in the whole department before the operation, and combined with the data, we initially thought that it was a benign lesion. When we communicated with the patient before surgery and explained the importance of preoperative needle biopsy, the patient decided not to do preoperative needle biopsy. We have to respect the patient's decision.

In this case, we chose a combination of iliac bone grafting and bone cement implantation. The reason is that the defect of the femoral lesion is large, and if only the ilium is used, a large amount of bone is required, which can destroy the structure of the pelvis. If there is a large amount of allogeneic bone to fill, it will greatly increase the medical cost. Therefore, we placed the obtained iliac bone close to the joint, which can increase the bone mass of the condyle after the fusion of the bone graft, and placed it with bone cement elsewhere. To increase the stability and strength of the femur after surgery, we chose a plate. Our recommendation for removal of the internal fixation device is based on the patient's own wishes. It is also worth noting that we only found CG in the femur, whether CG may exist in other parts is unknown. Whether further screening of other sites is required. Our consideration depends on two points, one is the patient's wishes and symptoms, and the other is to check the corresponding parts when the patient has symptoms. Because the incidence of CG is extremely low and slow. However, it is unclear whether systemic treatment is required to prevent future high cholesterol levels. Because of the current literature references, there is no correlation between CG and serum cholesterol levels. Therefore, there is no need to use drugs for prophylaxis, since this patient's current serum cholesterol level is normal. However, it is necessary to maintain good eating habits in daily life.

## Conclusion

We treated the disease for the first time *via* surgery and finally achieved satisfactory results. The reason for choosing surgery was because CG had already caused a certain amount of damage to the structure of the femur. However, CG, as a disease with a very low incidence, the most suitable treatment plan still needs further prospective studies.

## Data Availability

The original contributions presented in the study are included in the article/Supplementary Material, further inquiries can be directed to the corresponding author/s.
